# Return of Sound Production as a Biomarker of Bottlenose Dolphin Emergence from Anesthesia

**DOI:** 10.3390/ani13152531

**Published:** 2023-08-05

**Authors:** Brittany L. Jones, Abby M. McClain, Jessica J. Sportelli, Carolina Ruiz Le-Bert

**Affiliations:** 1 National Marine Mammal Foundation, 2240 Shelter Island Dr Ste 200, San Diego, CA 92106, USA; abby.mcclain@nmmf.org (A.M.M.); jessica.sportelli@nmmf.org (J.J.S.); 2 Naval Information Warfare Center Pacific, San Diego, CA 92107, USA; carolina.r.lebert.civ@us.navy.mil

**Keywords:** dolphin, acoustic, vocalization, anesthesia emergence, welfare

## Abstract

**Simple Summary:**

When humans are recovering from an anesthetic procedure, their medical care teams monitor a number of medical and behavioral biomarkers to ensure they are conscious and can safely return home from the hospital. For example, the ability of the patient to open their eyes, track objects, navigate their environment, speak, and respond appropriately to questions, are considered important supplemental behavioral biomarkers. We set out to monitor the return of sound production in bottlenose dolphins as they recovered following anesthesia. Sound production in dolphins is important for both their ability to navigate underwater using echolocation and to communicate with conspecifics using whistles. We found that otherwise healthy dolphins recovering from an anesthetic procedure produced echolocation clicks approximately 92 min following the return of spontaneous breathing. This return was correlated to the return of the righting reflex, or the dolphin’s ability to maintain balance in the water. Whistle production, used for communication, began after the emission of clicks. We suggest that underwater acoustic monitoring of bottlenose dolphins provides useful information to supplement other medical biomarkers of anesthetic recovery.

**Abstract:**

(1) Background: When a human or animal is recovering from general anesthesia, their medical team uses several behavioral and physiological parameters to assess their emergence from the unconscious state to complete wakefulness. However, the return of auditory and acoustic behaviors indicative of the complete return of consciousness in humans can be difficult to assess in a completely aquatic non-human mammal. Dolphins produce sound using the nasal system while using both passive auditory and active biological sonar (echolocation) to navigate and interrogate their environment. The sounds generated by dolphins, such as whistles and clicks, however, can be difficult to hear when the animal is submerged. (2) Methods: We implemented a system to audibly and visually (i.e., using spectrograms) monitor the underwater acoustic behavior of dolphins recovering from anesthesia. (3) Results: Eleven of the twelve recorded dolphins began echolocating within 92 min (Mean = 00:43:41 HH:MM:SS) following spontaneous respirations. In all cases, the dolphins echolocated prior to whistling (Mean = 04:57:47). The return of echolocation was significantly correlated to the return of the righting reflex (Mean = 1:13:44), a commonly used behavioral indicator of dolphin emergence. (4) Conclusions: We suggest that acoustic monitoring for the onset of click production may be a useful supplement to the established medical and behavioral biomarkers of restoring consciousness following anesthesia in bottlenose dolphins.

## 1. Introduction

Following the general anesthesia of human and non-human patients, a suite of neurobiological processes and interactions lead to the return of consciousness. Altogether, these events are known as the emergence period of anesthesia. Characterization of these biological processes, which begin following cessation of anesthetic delivery until the return of non-reflex responses to verbal commands, are lacking in many species [1,2]. Additionally, it is generally understood that this transition to consciousness cannot be completely explained by anesthetic drugs but also by molecular, genetic, and behavioral factors [3,4].

As in terrestrial species, anesthetic emergence in bottlenose dolphins (*Tursiops truncatus*) has yet to be well documented and understood. Empirical evidence supports an asymmetrical process (hysteresis) similar to that observed in human and non-human mammalian species [3]. Pharmacologic factors, such as central nervous system receptor binding and metabolism of anesthetic drugs and agents, as well as non-pharmacologic factors, such as body temperature regulation, electrolyte imbalances, and disease states, all likely contribute to the variations observed during anesthesia emergence. Although bottlenose dolphin anesthesia practices are outside of the scope of this manuscript, there is a rich history of dolphin anesthetic procedures dating back to the 1930s and continuing to the present [5,6,7,8,9,10,11,12]. Many of the same behavioral and physiological parameters used in human medicine and terrestrial veterinary medicine are preserved in dolphins. For example, the opening of the eyes, visual tracking, return of the palpebral and blink reflexes, return of oropharyngeal and lingual movements and jaw tone, and sometimes the return of spontaneous respirations are used to assess consciousness when determining readiness for removal of the endotracheal tube [9]. In addition, dolphin-specific reflexes monitored include the swimming reflex, observed as dorso-ventral undulations of the fluke, and blowhole tone and movement [9,10,13,14]. More recently, the restoration of normal individual cardiopulmonary features, such as stable mean arterial blood pressure and respiratory sinus arrhythmia, have been used.

Following the removal of the endotracheal tube and return of spontaneous ventilation, dolphins are usually moved to limited space enclosures for continued physiological and behavioral monitoring. Here, animal care teams can assess the return of righting reflexes and consciousness. The ability to maintain buoyancy and balance in the water column without aid, the use of pectoral flippers to navigate and remain upright, the use of the tail fluke for water propulsion, evidence of successful navigation of the environment, and appropriate responses to behavioral cues (i.e., a trained response to a hand signal given to the animal from their care team), all indicate that a dolphin has likely restored consciousness. Historically, these observations would indicate the end of the anesthetic emergence period, and the dolphin would be considered ready to safely return to their home enclosure or environment.

Bottlenose dolphins (hereafter referred to as dolphins) produce sound through the pressurization and muscular activation of paired nasal cavities (e.g., [15,16]). Unlike most terrestrial mammals, these sounds can be produced without overt physical movements. Therefore, sound production when a marine mammal is submerged can be difficult for humans to hear and/or visually identify. While dolphins do not have vocal cords and, therefore, produce phonations, we refer to any sound produced by the vocal apparatus of a dolphin as vocalization and use that term throughout for consistency with the previous literature [17,18]. Dolphins produce three main categories of vocalizations: clicks used for echolocation, burst pulses, and whistles [19]. Dolphins utilize underwater biological sonar (i.e., echolocation) to sense and navigate their environment. Echolocation is made up of emitted, broadband trains of clicks and their received echoes. Burst pulses are also made up of clicks that are emitted in short bursts or packets and at a fast rate (i.e., average inter-click interval = 0.004 s, [20]). These pulsed sounds are considered social communication signals and are typically recorded during agonistic and/or aggressive contexts (e.g., [21]). Burst pulse analyses were not included in the present study. Whistles are frequency and amplitude-modulated narrowband vocalizations that sound tonal to the human ear (e.g., [22,23]). Bottlenose dolphins develop and maintain individually distinctive whistle contours (i.e., patterns of frequency modulation over time) that are termed signature whistles (e.g., [22,24,25]). Signature whistles are most commonly emitted during periods spent isolated from conspecifics and are produced at an abnormally high repetition rate during periods of distress (e.g., [26,27,28,29,30]).

For the purposes of improving the health and welfare of bottlenose dolphins, the Sound and Health team of the National Marine Mammal Foundation endeavors to develop innovative tools for the monitoring and interpretation of acoustic behavior in dolphins. Sound production is imperative for dolphins to both safely navigate their environment and communicate their needs with conspecifics [31]. Thus, the return of sound production following general anesthesia of this species could be a useful biomarker in decision-making algorithms for program managers. Here, we describe the opportunistic application of an acoustic monitoring system in a group of bottlenose dolphins emerging from general anesthesia. The goals of this study were (1) to monitor the return of acoustic behavior during the emergence period and (2) to assess the utility of the return of acoustic behaviors as biomarkers for determining complete wakefulness in bottlenose dolphins.

## 2. Materials and Methods

### 2.1. Animals and Anesthetic Procedures

Bottlenose dolphins cared for by the U.S. Navy Marine Mammal Program, Naval Information Warfare Center Pacific, are housed in 9 m by 9 m floating, netted natural seawater enclosures in San Diego Bay, CA, USA. Over the period from January 2020 to February 2021, 10 healthy adult bottlenose dolphins (male = 6, female = 4; age range 8–46 years old) were placed under general anesthesia for oral, ophthalmic, renal, or pulmonary procedures (12 anesthetic procedures on 10 individual animals). Prior to each procedure, comprehensive physical examinations were performed by a veterinarian. As an index of general health and a prognostic tool for anesthesia-related complications, the physical status of individual dolphins was classified using the American Society of Anesthesiologists (ASA) physical status classification system [32,33]. Duration of anesthesia was defined as the difference between the time to intubation and the time to extubation (Table 1).

All dolphins were provided an intramuscular injection of midazolam, 0.08–0.1 mg/kg (Hospira, Inc., Lake Forest, IL, USA), combined with meperidine, 0.1–0.2 mg/kg (West-Ward Pharmaceuticals, Eatontown, NJ, USA). Induction of anesthesia was accomplished with intravascular midazolam, 0.02 mg/kg, and propofol, 1–4 mg/kg (Hospira, Inc., Lake Forest, IL, USA), to effect, for endotracheal intubation. Regional and/or local anesthesia of mandibular and/or maxillary nerves for oral procedures was accomplished using no more than 1 mg/kg of 2% lidocaine (Fresenius Kabi, Lake Zurich, IL, USA) for injection. Centralization of the eye during ophthalmic procedures was induced using injectable cis-atracurium for temporary paralysis (0.1 mg/kg every 15–20 min; Hospira, Inc., Lake Forest, IL, USA). All dolphins were maintained on a surgical plane of anesthesia using the volatile anesthetic gas sevoflurane (Piramal Critical Care, Inc., Bethlehem, PA, USA). Comprehensive physiologic monitoring was accomplished through electrocardiogram, pulse oximetry, direct blood pressure readings, capnography and respiratory gas analyses, and rectal temperature (GE Carescape B650, GE Healthcare, Finland), as well as arterial and venous blood gas analysis (ABL90 Flex; Radiometer Medical ApS, København, Denmark). Following completion of the medical or surgical procedure, reversal agents flumazenil, at a 1:13 reversal ratio (Fresenius Kabi, Lake Zurich, IL, USA) and naloxone, 10 mcg/kg (International Medication Systems Limited, El Monte, CA, USA), were administered intravenously. All dolphins recovered from anesthesia without complications.

### 2.2. Emergence from Anesthesia

Emergence from anesthesia was monitored and assessed in two phases using multiple physiological and behavioral parameters. The first phase ended with the successful removal of the endotracheal tube and replacement of the orally displaced larynx into the nasal cavities (i.e., extubation). The second phase ended with complete wakefulness and, therefore, veterinary-directed approval for dolphins to return to their natural seawater enclosures.

During the first phase of emergence, behavioral and physiological parameters were used to assess the transition to wakefulness. Behavioral parameters included the opening of the eyes and visual tracking, the return of palpebral and blink reflexes, gag reflex, and swimming reflex, the return of blowhole tone, and the return of oropharyngeal and lingual tone and movement. Physiological parameters included stable, unsupported mean arterial blood pressure, indicators of adequate oxygenation (i.e., normal mucosal membrane color and pulse oximetry readings), and the return of a normal respiratory sinus arrhythmia characteristic for this species. The return of spontaneous respirations was also used to assess emergence; however, not all dolphins spontaneously ventilated while intubated. Following successful extubation, dolphins were moved to a warm, shallow, above-ground seawater pool (constructed of laminated plastic; max depth = 1.5 m; diameter = 7 m) for phase two of anesthetic emergence. Dolphins were supported by personnel until the return of the righting reflex (i.e., the ability to maintain sternal positioning in water without support). However, further restoration of automatic and volitional behaviors, such as the ability to adjust buoyancy, tail fluke propulsion, proper pool navigation, and appropriate responses to behavioral cues, signaled complete wakefulness and restoration of consciousness. The time to return of the righting reflex was recorded in 11/12 anesthetic procedures and used as a pre-established comparative biomarker.

### 2.3. Acoustic Data Acquisition and Analysis

**Figure 1:**
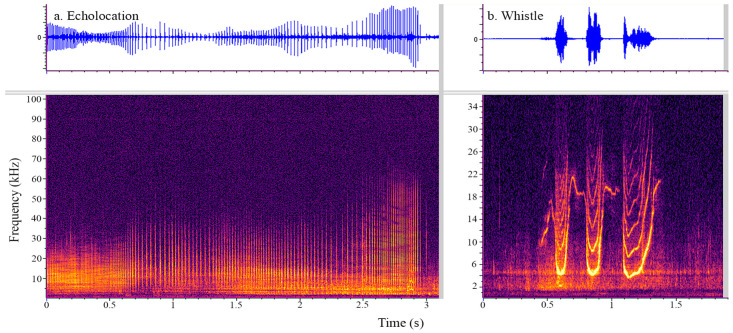
Spectrogram of low frequency clicks (**a**) and a whistle (**b**) produced by a bottlenose dolphi. Frequency (*x*-axis) is given in kHz, and time is presented in s (*y*-axis). The energy is given in a color gradation with lighter colors representing higher energy sound. The corresponding waveform (relative amplitude on the *y*-axis and time (s) on the *x*-axis) is plotted above the examples of echolocation clicks (panel (**a**)) and a whistle (panel (**b**)). Clicks appear as broadband vertical lines with little to no visible space (inter-click interval) between consecutive lines (clicks). The whistle can take many different shapes but appears as a bright narrow line that changes in frequency over time and can have harmonics (repetitive lines at interval frequencies above the lowest fundamental frequency contour).

Acoustic data were acquired during phase two of anesthetic emergence when the dolphin was isolated in the above-ground seawater pools. The acoustical system consists of a High Tech Inc (HTI) high-frequency hydrophone (2 Hz to 125 kHz, ±3 dB) that sits about a meter down in the water column, connected to a Behringer UMC202HD sound digitizing card. Real-time communication to a Panasonic CF-31 Toughbook laptop was accomplished via a USB interface. PAMGuard version 2.01.05 [34] software was used to create a live-feed visual spectrogram utilizing the following modules: sound acquisition, FFT, spectrogram display, National Marine Mammal Foundation Welfare Acoustic Monitoring system (NMMF WAMS) [35] sound recorder, and sound output (window type Hann, 192,000 Hz sample rate, Hop size 2048, 50% overlap, FFT length 4096, 0–39 kHz frequency range, 0–10 sec time window). A trained acoustic observer (B.L.J or J.J.S.) visually analyzed the live-feed recording throughout the recovery time (see Figure 1)). 

## 3. Results

**Figure 2:**
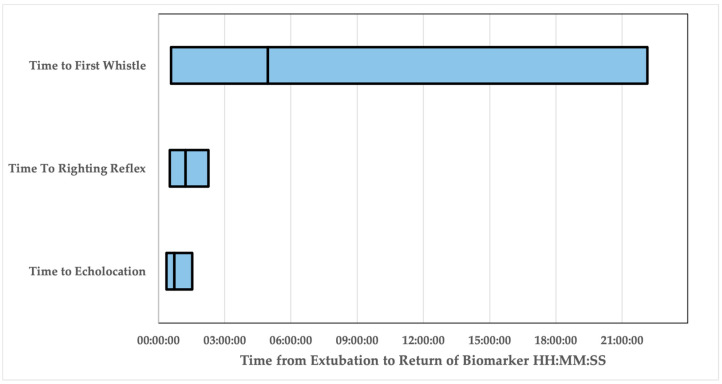
Bar graph depicting the range of time from extubation (HH:MM:SS, *x*-axis) to the time of return of the biomarkers explored. The start of the bar represents the minimum time from extubation the biomarker was recorded, and the end of the bar represents the maximum time from extubation for this group of dolphins. The vertical line within each bar demonstrates the mean.

The time to return of the righting reflex (Mean (M) = 01:13:44 HH:MM:SS, Standard Error (SE) = 00:09:02; Range = 00:31:00–02:16:00) following extubation was used as a previously established biomarker of anesthetic emergence. The time to return of echolocation (M = 00:43:41, SE = 00:06:28, Range 00:21:58–01:32:09) and the time to whistle (M = 04:57:47, SE = 02:22:31, Range 00:28:57–22:08:08) following extubation were assessed as novel biomarkers of emergence (Table 2, Figure 2). In 9/12 events, dolphins recorded whistling during the emergence period and began whistling only after echolocating.

Non-parametric correlation tests were performed between the three variables to assess relatedness among biomarkers. The time to echolocation was significantly correlated with the time to return of the righting reflex (Spearman’s rho = 0.648, *p* = 0.043). Time to whistle was not significantly related to either time to echolocation (Spearman’s rho = 0.617, *p* = 0.077) or time to return of the righting reflex (Spearman’s rho = 0.619, *p* = 0.102).

## 4. Discussion

Here, we provide preliminary information on the return of sound production as an adjunctive biomarker of bottlenose dolphin anesthetic emergence. Time to echolocation correlated with the return of the dolphin righting reflex—a reflex indicating the restoration of visual, vestibular, and somatosensory input. The correlation and return of these two biomarkers suggest the dolphin CNS is no longer inhibited by anesthetic agents, and restoration of consciousness and complete wakefulness are achieved. Similarly, a medical team monitoring the emergence from general anesthesia in humans considers both physiological and behavioral indicators of restoration of consciousness following anesthesia-induced unconsciousness. Two imperative behavioral indicators are the ability to navigate the environment using vision and the ability to speak and respond to questions appropriately. As echolocation is the dolphin’s primary sensory modality for navigation [31,36], we suggest that the return of echolocation following anesthesia could be a useful biomarker for program managers to ensure a dolphin is capable of navigating its environments, especially in low light or visually occluded waters. Due to the observed individual differences, non-standardized anesthetic drug protocols, complex interactions of the physiology of anesthesia, and the neurobiological mechanisms underlying the anesthetic emergence phase, our recommendation is to utilize acoustic monitoring as an adjunctive biomarker of restoration of consciousness in dolphins. Such acoustic biomarkers could be used as supplemental indicators of anesthetic emergence and should not be considered a replacement for other critical physiologic and behavioral indicators.

In the current study, we did not find that whistles were a consistent and useful cue to indicate anesthetic emergence. Whistles began after echolocation, if at all, and showed much more variability in their time to and rate of production. Similarly, humans recovering from anesthesia typically open their eyes and scan their environment prior to attempting to communicate vocally. Whistle production was not significantly correlated to the return of the righting reflex biomarker, nor was it correlated with the return of echolocation. Whistles are used by dolphins primarily for communication with conspecifics. However, dolphins who are in distress produce their signature whistle at a high repetition rate and intensity. It is unclear if the time to whistle production could be considered a delay in anesthetic emergence, and, if so, if it could be a result of reduced anesthetic drug metabolism or clearance, the lack of conspecifics in proximity making communication superfluous, the lack of stress or anesthetic complications (i.e., distress whistling was unnecessary), or a combination of all of these factors. Future studies should quantify dolphin whistle behavior during control contexts to assess whether individual differences in whistle production were related to a dolphin’s propensity to whistle. Whether the whistle characteristics change after an anesthetic procedure, and if so, when they return to baseline, are also interesting questions for future studies.

All dolphins in this study received comprehensive pre-anesthetic health assessments, including blood sample analysis, physical examination, thoracic and abdominal ultrasound, electrocardiogram assessment, and, in a few cases, echocardiogram. Dolphins were then categorized into a physical status category, as described by the American Society of Anesthesiologists (ASA), for determining anesthetic risk based on health indicators. While this method of categorizing health is utilized in both human and veterinary medicine, subjectivity could account for inaccuracies in method implementation [32,33]. It is important to note that previous studies suggest that a high whistle rate could be an indicator of poor health status (e.g., [26,27,28,29,30]). Thus, in critical care cases, where dolphins may have significant disease prior to anesthesia, it is feasible that whistle behavior changes could be an indicator of distress and should not be ignored.

To assess complete wakefulness, humans recovering from anesthesia are commonly asked to respond to simple questions, such as ‘What is your name?’. Similarly, future efforts could emphasize training dolphins to respond to hand signal cues using their individual signature whistles. Capturing this behavior would allow animal care teams to request the signature whistle from an individual dolphin, thereby not only assessing the dolphin’s ability to respond to hand signals but also confirming memory and implementation of learned behavior and the return of motor control of the blowhole and sound production apparatus. Specifically, eliciting the individual’s signature whistle further demonstrates the dolphin’s ability to produce a distress call, which could elicit helping behaviors from conspecifics if needed following recovery from anesthesia [30].

As the present report was prepared as an opportunistic application, it provides an interesting foundation for future research to build upon. In other words, to provide robust validation of biomarkers of anesthetic emergence in dolphins, formal assessments and comparisons of medical, pharmacologic, physiologic, behavioral, and acoustic variables in large sample sizes are warranted. Variables for future study consideration include, but should not be limited to, sex and age categories, genetic factor categories, time of day of the sound production study, and overall physical status to evaluate for possible interaction effects on time to echolocate following general anesthesia in this species.

While we were able to confirm that dolphins in this study regained the ability to produce clicks, future work should attempt to characterize whether dolphins are successfully utilizing the echoes of produced clicks to navigate their environment. One such strategy could involve the implementation of physical barriers to assess successful echo utilization in navigation. The combination of echolocation production, followed by successful navigation away from an obstacle, may provide additional assurances that dolphins are successfully receiving and integrating information from the self-produced sound.

## 5. Conclusions

In conclusion, the return of spontaneous echolocation in dolphins could be a useful supplemental indicator of restoration of consciousness and complete wakefulness in dolphins recovering from general anesthesia. Specifically, the production of sound and the use of echolocation could be useful biomarkers in decision-making algorithms for program managers to determine the appropriate cues for returning dolphins back to unassisted, free-swimming environments. Future studies focused on increased sample sizes, controlled pharmacologic and physiologic studies, and assessment of acoustic behavioral indicators in dolphins experiencing prolonged anesthetic emergence will be necessary for generalizing to the greater MMP dolphin population and beyond. As such, the acoustic behavior of aquatic animals is an underutilized indicator in aquatic medicine compared to terrestrial animal or human medicine. Therefore, we recommend that animal care managers strongly consider sound production when developing health and welfare monitoring programs for bottlenose dolphins under their care.

## Figures and Tables

**Table 1:** **Focal animal characteristics.** Sex, age, medical, or surgical procedure requiring general anesthesia, American Society of Anesthesiologists (ASA) physical status classification score (I–VI), and duration of anesthesia for each dolphin. * Denotes the same individual dolphin anesthetized on two separate occasions. ^ Denotes a second dolphin anesthetized on two separate occasions.

Dolphin	Sex	Age	Medical or Surgical Procedure	ASA Classification	Duration of Anesthesia (HH:MM)
1 *	F	37	Dental extractions	II	03:37
2	M	8	Bronchoscopy	II	03:41
3	M	29	Dental extractions	II	04:33
4 ^	F	45	Corneal graft	II	00:56
5 *	F	38	Dental extractions	II	03:07
6	M	42	Dental extractions	II	03:34
7	F	16	Dental extractions	II	02:58
8	F	40	Dental extractions	II	03:16
9	M	41	Corneal graft	II	00:30
10	M	10	Bronchoscopy	II	04:03
11	M	37	Dental extractions	II	03:39
12 ^	F	46	Ureteroscopy	IV	03:08

**Table 2:** **Descriptive statistics for biomarker variables assessed during emergence from general anesthesia**. The minimum time to, the maximum time to, the mean time to, and the standard error for the mean time to are reported for time to echolocate, time to the return of the righting reflex, and time to whistle. All times are given in HH:MM:SS format.

	Time to Echolocation	Time to Return of Righting Reflex	Time to Whistle
Dolphins	11/12	11/12	9/12
Minimum	00:21:58	00:31:00	00:28:57
Maximum	01:32:09	02:16:00	22:08:08
Mean	00:43:41	01:13:44	04:57:47
Standard Error	00:06:28	00:09:02	02:22:31

## Data Availability

The datasets generated during and/or analyzed during the current study are not publicly available. Data may be made available upon approval of a formal sample request to the MMP. The PAMGuard is freely available for download at https://www.pamguard.org/. The custom NMMF Welfare Acoustic Monitoring System is currently publicly available at Pamguard Plugins.
